# Microbial extracellular electron transfer and its relevance to iron corrosion

**DOI:** 10.1111/1751-7915.12340

**Published:** 2016-02-10

**Authors:** Souichiro Kato

**Affiliations:** ^1^ Bioproduction Research InstituteNational Institute of Advanced Industrial Science and Technology (AIST)2‐17‐2‐1 Tsukisamu‐HigashiToyohira‐kuSapporoHokkaido062‐8517Japan; ^2^ Research Center for Advanced Science and TechnologyThe University of Tokyo4‐6‐1 KomabaMeguro‐kuTokyo153‐8904Japan; ^3^ Division of Applied BioscienceGraduate School of AgricultureHokkaido UniversityKita‐9 Nishi‐9Kita‐kuSapporoHokkaido060‐8589Japan

## Abstract

Extracellular electron transfer (EET) is a microbial metabolism that enables efficient electron transfer between microbial cells and extracellular solid materials. Microorganisms harbouring EET abilities have received considerable attention for their various biotechnological applications, including bioleaching and bioelectrochemical systems. On the other hand, recent research revealed that microbial EET potentially induces corrosion of iron structures. It has been well known that corrosion of iron occurring under anoxic conditions is mostly caused by microbial activities, which is termed as microbiologically influenced corrosion (MIC). Among diverse MIC mechanisms, microbial EET activity that enhances corrosion via direct uptake of electrons from metallic iron, specifically termed as electrical MIC (EMIC), has been regarded as one of the major causative factors. The EMIC‐inducing microorganisms initially identified were certain sulfate‐reducing bacteria and methanogenic archaea isolated from marine environments. Subsequently, abilities to induce EMIC were also demonstrated in diverse anaerobic microorganisms in freshwater environments and oil fields, including acetogenic bacteria and nitrate‐reducing bacteria. Abilities of EET and EMIC are now regarded as microbial traits more widespread among diverse microbial clades than was thought previously. In this review, basic understandings of microbial EET and recent progresses in the EMIC research are introduced.

## Introduction

Acquisition of energy is an indispensable activity for all living organisms. Most organisms, including human beings, conserve energy through respiration, with organic compounds and oxygen gas as the electron donor and acceptor respectively. In contrast, many microorganisms have the ability to utilize diverse inorganic compounds as the substrates for respiration. Furthermore, some particular microorganisms have the ability to acquire energy through transferring electrons to or from extracellular solid compounds. This microbial metabolism is specifically termed as ‘extracellular electron transfer (EET)’ (Gralnick and Newman, 2007; Richter *et al*., 2012). In addition to naturally occurring metal minerals, microorganisms harbouring EET abilities can utilize artificial conductive materials, including graphite and metal electrodes, as the electron donor or acceptor (Bond and Lovley, 2003; Gregory *et al*., 2004). This microbial activity has received considerable attention for biotechnological applications, including bioremediation of toxic metals and diverse bioelectrochemical systems (Arends and Verstraete, 2012; Logan and Rabaey, 2012; Kato, 2015). Besides, recent studies have disclosed that some microorganisms utilize zero‐valent metallic iron as their electron donor via EET metabolisms, which stimulates iron corrosion under anoxic conditions. This review introduces the basic knowledge in microbial EET metabolisms and the recent research progresses on the relevance of microbial EET to iron corrosion.

## Microbial EET

**Figure 1:**
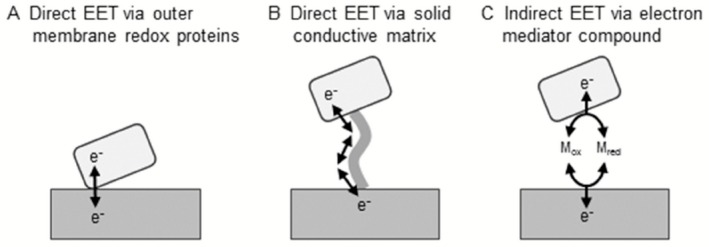
The schematic images of three microbial EET mechanisms. A. Direct EET via outer membrane redox proteins. B. Direct EET via solid conductive matrix (e.g. conductive pili). C. Indirect EET via an electron mediator compound (M_red_/M_ox_).

As for commonly characterized energy metabolisms, microorganisms first must incorporate substrates for respiration into their cells, where oxidation and reduction of substrates proceed. In contrast, special molecular mechanisms are required for EET‐based energy metabolisms, since microorganisms cannot incorporate solid materials into their cells. Mechanisms of microbial EET can be categorized into either direct or indirect manners. In the direct EET, microorganisms attach to solid surfaces, to or from which they directly transfer electrons (Fig. 1A). The molecular mechanisms for the direct EET have been intensively investigated for some iron‐reducing and iron‐oxidizing bacteria, including *Geobacter sulfurreducens*,* Shewanella oneidensis* and *Acidithiobacillus ferrooxidans* (Stams *et al*., 2006; Weber *et al*., 2006; Shi *et al*., 2007; Castelle *et al*., 2008). These microorganisms utilize metal‐containing redox proteins, such as *c*‐type cytochromes and rusticyanin, to electrically connect intracellular respiratory chains and extracellular solid materials. Furthermore, *G. sulfurreducens* and *S. oneidensis* were reported to produce conductive filamentous apparatus (pili and outer membrane extensions, respectively) that are specifically termed as ‘nanowires’ (Reguera *et al*., 2005; Gorby *et al*., 2006; Pirbadian *et al*., 2014). These microorganisms have the ability to transfer electrons to or from distantly located solid materials using the filaments as ‘electric wire’ (Fig. 1B).

In contrast, some microorganisms indirectly transfer electrons to or from solid compounds using diffusible redox chemicals, referred to as electron mediators (Watanabe *et al*., 2009). Microorganisms reduce (or oxidize) intracellular electron mediators, after which the reduced (or oxidized) electron mediators diffuse out of the cell to solid surfaces and donate (or accept) electrons, and then the oxidized (or reduced) mediators return back into the cells and are again utilized as respiratory substrates (Fig. 1C). Some microorganisms have the ability to synthesize low‐molecular‐weight organic compounds that work as electron mediators, including phenazine compounds and flavin derivatives (Rabaey *et al*., 2004; Marsili *et al*., 2008). Naturally occurring (e.g. humic substances) and artificial (e.g. quinone derivatives, ferrocene derivatives) redox chemicals can also work as electron mediators to propel microbial EET (Jiang and Kappler, 2008; Watanabe *et al*., 2009; Nishio *et al*., 2013). The reader is referred to several excellent review articles on the mechanisms of direct and indirect EET (Hernandez and Newman, 2001; Shi *et al*., 2007; Lovley, 2008; Thrash and Coates, 2008; Watanabe *et al*., 2009; Richter *et al*., 2012).

**Table 1:** Isolated strains identified as EMIC‐inducing microorganisms

Metabolic group Strain name	Isolation sources	Phylogeny		References
		Phylum/class	Family	
Sulfate‐reducing bacteria				
‘*Desulfopila corrodens*’ IS4	Marine sediment	*Deltaproteobacteria*	*Desulfobulbaceae*	Dinh and colleagues (2004)
‘*Desulfovibrio ferrophilus*’ IS5	Marine sediment	*Deltaproteobacteria*	*Desulfovibrionaceae*	Dinh and colleagues (2004)
Methanogenic archaea				
*Methanobacterium* sp. IM1	Marine sediment	*Euryarchaeota*	*Methanobacteriaceae*	Dinh and colleagues (2004)
*Methanococcus maripaludis* KA1	Crude‐oil storage tank	*Euryarchaeota*	*Methanococcaceae*	Uchiyama and colleagues (2010)
*Methanococcus maripaludis* Mic1c10	Crude‐oil storage tank	*Euryarchaeota*	*Methanococcaceae*	Mori and colleagues (2010)
Acetogenic bacteria				
*Sporomusa* sp. GT1	Rice paddy field	*Firmicutes*	*Veillonellaceae*	Kato and colleagues (2015)
Nitrate‐reducing bacteria				
*Bacillus licheniformis* ATCC 14580	Soil	*Firmicutes*	*Bacillaceae*	Xu and colleagues (2013)
*Prolixibacter* sp. MIC1‐1	Crude‐oil well	*Bacteroidetes*	*Prolixibacteraceae*	Iino and colleagues (2015)

**Figure 2:**
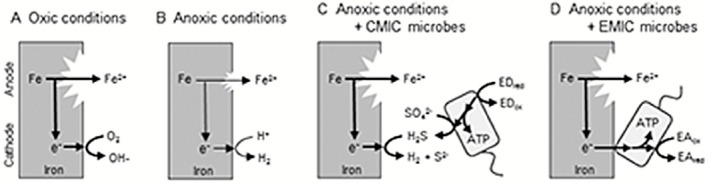
The schematic images of the iron corrosion mechanisms. Spontaneous, oxidative dissolution of metallic iron into ferrous iron (the anodic reaction) is the first step of corrosion in all of the cases. A. Under oxic conditions, electrons derived from iron oxidation are consumed by reduction of O_2_ (the cathodic reaction). B. Under anoxic conditions, the possible cathodic reaction is the reduction of H ^+^ into H_2_, while the corrosion rate is quite low. C. CMIC‐inducing SRB reduce sulfate into corrosive H_2_S, which stimulate the cathodic reaction and hence iron corrosion. In this case, SRB requires exogenous electron donors (ED, such as lactate and H_2_) to reduce sulfate and acquire energy. D. EMIC‐inducing microorganisms directly utilize electrons in metallic iron as the electron donor to stimulate iron corrosion. Sulfate reduction, methanogenesis, acetogenesis and nitrate reduction were reported as the electron‐accepting reactions (EA_red_/EA_ox_).

**Figure 3:**
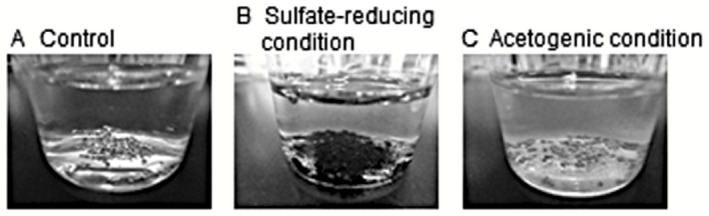
Corrosion products formed by EMIC‐inducing microorganisms. A. Non‐inoculated control. B. An iron‐corroding enrichment culture under sulfate‐reducing conditions. C. An iron‐corroding enrichment culture under acetogenic conditions.

## Microorganisms and corrosion of iron

Corrosion of iron is an electrochemical process consisting of oxidation of metallic iron to ferrous ion (anodic reaction, Eq. (1)) and reduction of electron acceptor compounds (cathodic reaction):

(1) $${\text{Fe}}^{2+}+2e^{-}↔\text{Fe}\;(0);\;E_{0}^{pc\prime }=-0.47\;V,$$

where −0.47 V references the standard hydrogen electrode (the same applies hereafter). In oxic environments, the cathodic reaction is reduction of oxygen (Eq. (2)) and corrosion rate is relatively high (Fig. 2A):

(2) $$O_{2}+4e^{-}+2H_{2}O↔4{\text{OH}}^{-};\;E_{0}^{pc\prime }=+0.82\;V.$$

In contrast, the most common electron consuming reaction under anoxic condition is proton reduction to hydrogen gas (Eq. (3)) (Fig. 2B):

(3) $$2H^{+}+2e^{-}↔H_{2};\;E_{0}^{pc\prime }=-0.41\;V.$$

Theoretically, iron corrosion in anoxic environments does not become a serious problem, since the proton reduction reaction on iron surfaces is usually a particularly slow reaction. However, iron corrosion under anoxic conditions has often been reported, and in most cases it is thought to be mediated by microbial metabolic activities (Lee *et al*., 1995; Hamilton, 2003; Beech and Sunner, 2004; Videla and Herrera, 2005).

Corrosion of iron caused by microbial metabolism is specifically termed as microbiologically influenced corrosion (MIC) or biocorrosion. Microbiologically influenced corrosion under anaerobic conditions often damages energy infrastructures, including crude oil reservoir tanks and underground oil and gas pipelines, causing enormous economic losses. The microorganisms first identified as MIC‐inducers were sulfate‐reducing bacteria (SRB) (Gaines, 1910). Sulfate‐reducing bacteria produce hydrogen sulfide through reduction of sulfate (Eq. (4)) with various organic compounds and often H_2_ as the source of reducing equivalents:

(4) $${\text{SO}}_{4}^{2-}+10H^{+}+8e^{-}↔H_{2}S+4H_{2}O;\;E_{0}^{pc\prime }=-0.22\;V.$$

Hydrogen sulfide is a highly oxidative chemical and is known to react with metallic iron to generate iron sulfide (Eq. (5)):

(5) $$H_{2}S+{\text{Fe}}^{0}\to \text{FeS}+H_{2}.$$

This process enhances corrosion of iron (Fig. 2C). Similarly, other microorganisms that secrete corrosive chemicals such as organic acids also stimulate corrosion of iron (Little *et al*., 2001; Usher *et al*., 2014). This manner of MIC, i.e. stimulation of the cathodic reaction by microbial metabolic end‐products, is referred to as chemical MIC (CMIC; Enning *et al*., 2012).

In contrast, acceleration of cathodic reactions via consumption of cathodic electrons, often referred to as cathodic depolarization, had been believed as one of the most prominent causes of MIC (Wolzogen Kühr, 1961). Microorganisms consuming H_2_ abiotically generated on iron surfaces (Eq. (3)) coupled with sulfate reduction (Eq. (4)) or methanogenesis (Eq. (6)) were considered to induce cathodic depolarization (King and Miller, 1971; Daniels *et al*., 1987):

(6) $${\text{HCO}}_{3}^{-}+9H^{+}+8e^{-}↔{\text{CH}}_{4}+3H_{2}O;\;E_{0}^{pc\prime }=-0.24\;V.$$

However, stimulation of anaerobic corrosion via H_2_ consumption has been viewed critically (Hardy, 1983), and recent studies revealed that H_2_ consumption itself is not sufficient to induce fatal iron corrosion as often observed in actual fields (Dinh *et al*., 2004; Mori *et al*., 2010). These studies implied that only microorganisms with EET ability, which enables efficient uptake of electrons from metallic iron, can stimulate the cathodic reaction and hence iron corrosion (Fig. 2D). This manner of MIC, i.e. stimulation of the cathodic reaction by consuming cathodic electrons as the metabolic energy sources, is referred to as electrical MIC (EMIC) (Enning *et al*., 2012). In the following sections, specific examples of microorganisms causing EMIC are introduced and their putative EET mechanisms are discussed.

## Microorganisms inducing EMIC

So far microorganisms in four different metabolic groups (SRB, methanogenic archaea, acetogenic bacteria and nitrate‐reducing bacteria) are elucidated as EMIC‐inducers (Table 1). In this section, specific examples of the EMIC‐inducing microorganisms are introduced.

### SRB

As introduced in the previous section, SRB is known as one of the most prominent bacteria to cause anaerobic MIC, mainly via production of corrosive chemical H_2_S. In addition to the indirect mechanisms, recent research disclosed that some SRB strains stimulate corrosion via more direct manners. Dinh and colleagues (2004) reported enrichment and isolation of two novel SRB strains from marine sediment using metallic iron as the sole electron donor. These isolated strains, subsequently denominated as ‘*Desulfopila corrodens*’ strain IS4 and ‘*Desulfovibrio ferrophilus*’ strain IS5 (Enning *et al*., 2012), reduced sulfate with concomitant oxidation of metallic iron much faster than abiotic H_2_ generation in an organic matter‐free medium. In contrast, the closely related SRB with efficient H_2_‐utilizing abilities did not show such activities (Dinh *et al*., 2004; Gittel *et al*., 2010). So far, EMIC‐inducing abilities in SRB have been identified in only a limited number of strains in the deltaproteobacterial families *Desulfovibrionaceae* and *Desulfobulbaceae* (Enning and Garrelfs, 2014). These reports suggest that the EMIC‐inducing SRBs have special molecular mechanisms for electron uptake from metallic iron, and the ability is a species and/or strains‐specific trait rather than universal in SRB. Under sulfate‐reducing conditions, black mineral crust mainly consisting of iron sulfide minerals is generally formed as corrosion products (Enning *et al*., 2012) (Fig. 3).

### Methanogenic archaea

Methane production by methanogenic archaea (Eq. (6)) is a major microbial metabolism in anoxic environments, especially in sulfate‐depleted zones. Participation of microbial methanogenesis to cathodic depolarization and anaerobic MIC was proposed in the 1980s (Daniels *et al*., 1987). The first direct evidence for an EMIC‐inducing methanogen was reported by Dinh and colleagues (2004). A new methanogenic strain, *Methanobacterium* sp. strain IM1, isolated from marine sediment, was shown to grow and produce methane with metallic iron as the sole electron source, and stimulate corrosion of iron (Dinh *et al*., 2004). Another research group in Japan also isolated EMIC‐inducing methanogenic strains, namely *Methanococcus maripaludis* strain KA1 and strain Mic1c10, from crude oil reservoir tanks (Mori *et al*., 2010; Uchiyama *et al*., 2010). Interestingly, authentic hydrogenotrophic methanogens closely related to these EMIC‐inducing methanogens did not grow well on and produce methane from metallic iron, indicating that the corrosive ability is a limited trait for certain methanogenic archaea.

### Acetogenic bacteria

Acetogenic bacteria have the abilities to conserve energy via the reductive acetyl‐CoA pathway, by which acetate is produced through reduction of carbon dioxide (Eq. (7)):

(7) $$2{\text{HCO}}_{3}^{-}+9H^{+}+8e^{-}↔{\text{CH}}_{3}{\text{COO}}^{-}+4H_{2}O;\;E_{0}^{pc\prime }=-0.28\;V.$$

Acetogenic bacteria generally utilize hydrogen gas or certain organic compounds as the source of reducing equivalents. While engagement of microbial acetogenesis for anaerobic MIC in low‐sulfate environments was postulated (Suflita *et al*., 2008), the direct evidence had not been demonstrated. Recently, Mand and colleagues (2014) demonstrated acetate production in microbial enrichment cultures with metallic iron as the sole electron source, from which bacteria closely related to known acetogenic bacterium (*Acetobacterium* sp.) were detected. Our research group also enriched acetogenic microbial communities with metallic iron as the sole energy source (Kato *et al*., 2015). The enriched communities produced acetate coupled with oxidation of metallic iron and produced significantly larger amount of ferrous iron than the abiotic controls. We eventually isolated *Sporomusa* sp. strain GT1 from the enrichments and demonstrated that the isolate acetogenetically grew on metallic iron and enhanced iron corrosion, while other well‐known acetogenic bacteria did not. In addition to the direct effects, the metabolism of acetogenic bacteria should cause another peripheral effects on iron corrosion. Most SRB cannot grow autotrophically and require organic compounds such as acetate as the carbon source. Hence, it is postulated that acetate generated by acetogenic bacteria indirectly enhances iron corrosion via stimulation of growth of CMIC‐ and/or EMIC‐inducing SRB (Mand *et al*., 2014).

### Nitrate‐reducing bacteria (NRB)

Reduction of nitrate (Eq. (8)) is also a major microbial metabolism under anoxic conditions:

(8) $${\text{NO}}_{3}^{-}+2H^{+}+2e^{-}↔{\text{NO}}_{2}^{-}+H_{2}O;\;E_{0}^{pc\prime }=+0.44\;V.$$

Recent studies revealed that particular NRB also causes EMIC. For instance, Xu and colleagues (2013) demonstrated that *Bacillus licheniformis* ATCC 14580, a facultative NRB strain, stimulates corrosion of carbon steel under nitrate‐reducing conditions. Iino and colleagues (2015) isolated another EMIC‐causing NRB, *Prolixibacter* sp. strain MIC1‐1, which is the first corrosive representative belonging to the phylum *Bacteroidetes*. In addition to the ability of *Prolixibacter* sp. strain MIC1‐1 to utilize cathodic electrons in metallic iron as the electron donor, the metabolic end‐product, nitrite, which is known as a strong corrosive compound (Lin *et al*., 2008), appears to induce severe corrosion (Iino *et al*., 2015). Nitrate injection into oil and gas reservoirs has been used for mitigation of souring and MIC caused by SRB, since it promotes growth of NRB and in turn suppresses growth of SRB (Gieg *et al*., 2011). However, the discovery of MIC‐inducing NRB led to the revelation that supplementation of nitrate may risk inducing NRB‐assisted corrosion. In contrast to sulfate‐reducing conditions, greyish mineral crust mainly containing iron carbonate and iron phosphate is generally formed as corrosion products under methanogenic, acetogenic and nitrate‐reducing conditions (Uchiyama *et al*., 2010; Iino *et al*., 2015) (Fig. 3).

### How do corrosive microorganisms uptake electrons from metallic iron?

The EMIC‐inducing microorganisms should have either direct or indirect EET abilities to efficiently uptake electrons from metallic iron. Physiological and electrochemical analyses have disclosed that the corrosive SRB strains (*Desulfopila corrodens* strain IS4 and *Desulfovibrio ferrophilus* strain IS5) appear to directly uptake electrons from metallic iron (Enning *et al*., 2012; Venzlaff *et al*., 2013). Production of the mineral crust (containing FeS, FeCO_3_ and Mg/CaCO_3_) with semiconductor‐like properties by the EMIC‐inducing SRB also supports this assumption. Deng and colleagues (2015) electrochemically demonstrated that the corrosive SRB *Desulfovibrio ferrophilus* strain IS5 is capable of extracting electrons from an inert electrode without consuming H_2_ as an electron carrier. Similarly, Beese‐Vasbender and colleagues (2015) reported that the corrosive methanogen *Methanobacterium* sp. strain IM1 generates cathodic current and produces methane at an electrode potential of −0.4 V, which is much more positive than the potential required for H_2_ production on a graphite cathode. Furthermore, Iino and colleagues (2015) confirmed that the *Prolixibacter* sp. strain MIC1‐1 does not have ability to oxidize H_2_, while this strain showed high EMIC activities coupled with nitrate reduction. These studies indicate that at least a part of EMIC‐causing microorganisms have the abilities for direct EET, while the molecular mechanisms remain unknown.

There are some reports on the relevance of indirect EET to EMIC. In the indirect EET, electron mediator molecules are required to achieve electron transfer from metallic iron to microbial cells. Zhang and colleagues (2015) demonstrated that exogenous addition of electron mediators [riboflavin and flavin adenine dinucleotide (FAD)] accelerates corrosion of stainless steel by *Desulfovibrio vulgaris*. Some EET‐harbouring microorganisms such as *Shewanella* spp. were reported to produce and excrete riboflavin and FAD to facilitate their EET activities (Marsili *et al*., 2008; Brutinel and Gralnick, 2012). These findings suggest that electron mediators secreted by microorganisms themselves facilitate EMIC, although there has been no direct evidence yet. Naturally occurring inorganic chemicals are another candidate of electron mediators facilitating EMIC. Manganese‐oxidizing bacteria were reported to conduct this type of corrosion (Dickinson *et al*., 1997; Ashassi‐Sorkhabi *et al*., 2012). In this case, oxidized forms of manganese [Mn(III/IV)] consume cathodic electrons in metallic iron and is reduced to Mn(II), which is oxidized back to Mn(III/IV) by manganese‐oxidizing bacteria. Iodide‐oxidizing bacteria isolated from brine in an iodide production facility stimulate iron corrosion in a similar manner with redox coupling of I^−^/I_2_ (Wakai *et al*., 2014).

Hydrogen gas is also a possible candidate of an electron mediator molecule. As discussed above, however, a number of studies showed that microorganisms that simply consume H_2_ do not induce significant corrosion of iron. Recently, Deutzmann and colleagues (2015) demonstrated a novel mechanism for electron uptake from metallic iron with hydrogen gas (and possibly formate) as an important intermediate. They discovered that cell‐free spent culture medium of a corrosive methanogen *Methanococcus maripaludis* strain MM901 accelerated H_2_ (and formate) generation from metallic iron, while that of a hydrogenase‐deficient mutant did not. This study suggests that surface‐associated redox enzymes, such as hydrogenases and formate dehydrogenase, are sufficient to mediate an apparent electron uptake that enhances iron corrosion.

The EET mechanisms for EMIC remains controversial, and it may be highly possible that each EMIC‐inducing microorganism utilizes completely different EET strategies. Also in the research field of bioelectrochemistry, the molecular mechanisms of microbial electron uptake from cathodes are much more enigmatic rather than the opposite reaction, i.e. microbial electron injection to anodes (Rosenbaum *et al*., 2011). Further research with multiple viewpoints, including microbial physiology, genomics, molecular biology and electrochemistry, is required to understand how EMIC‐inducers efficiently uptake electrons from metallic iron.

## Concluding remarks

The studies on MIC in the last decades have demonstrated that physiologically and phylogenetically diverse microorganisms, including SRB (*Proteobacteria*), methanogens (*Euryarchaeota*), acetogens (*Firmicutes*) and NRB (*Firmicutes* and *Bacteroidetes*), stimulate iron corrosion via their EET metabolisms. Further investigation on these microorganisms, especially on their EET mechanisms, and also identification of novel EMIC‐causing microorganisms will lead to technological development for MIC mitigation. In addition, EMIC‐causing microorganisms can potentially be utilized as novel biocatalysts. Microbial electron uptake from solid materials is being pursued as biocatalysts for cathode reactions in diverse microbial electrochemical systems (Rabaey and Rozendal, 2010; Erable *et al*., 2012; Kim *et al*., 2015). Based on this idea, cathodic methane production by a corrosive methanogen has already been examined (Beese‐Vasbender *et al*., 2015). Close relatives of a corrosive acetogen are also being investigated as biocatalysts for conversion on electrical energy into liquid fuels (Nevin *et al*., 2011).

Considering that almost all metallic iron has been recently introduced into environments by human activities, EMIC reaction, namely utilization of metallic iron as an electron donor of respiration, is an enigmatic metabolism from the viewpoint of evolution. One plausible explanation is that EMIC reaction is due to the promiscuous usage of other analogous metabolisms. Our group recently demonstrated that electrically conductive metal minerals, which abundantly exist in natural environments (Hochella *et al*., 2008; Nakamura *et al*., 2010a), serve as an electron source and sink for some EET‐harbouring microorganisms (Nakamura *et al*., 2009; 2010b; Kato *et al*., 2010, 2013). Furthermore, a novel microbial symbiotic metabolism, in which electrons released by one microorganism are transferred to another via electric current flowing through biological and mineralogical solid materials, has recently been demonstrated (Summers *et al*., 2010; Kato *et al*., 2012a). Diverse kinds of microorganisms, including NRB, ferric iron reducers, methanogens and dehalorespirators, have been reported to participate in the electron‐consuming part of the symbiotic process (Summers *et al*., 2010; Kato *et al*., 2012a,b; Aulenta *et al*., 2013). Further studies on EMIC will also give novel insights for ecological and evolutional aspects of microbial EET.

## Conflict of Interest

None declared.

## References

[mbt212340-bib-0001] Arends, J.B. , and Verstraete, W. (2012) 100 years of microbial electricity production: three concepts for the future. Microb Biotechnol 5: 333–346. 21958308 10.1111/j.1751-7915.2011.00302.x PMC3821677

[mbt212340-bib-0002] Ashassi‐Sorkhabi, H. , Moradi‐Haghighi, M. , and Zarrini, G. (2012) The effect of *Pseudoxanthomonas* sp. as manganese oxidizing bacterium on the corrosion behavior of carbon steel. Mater Sci Eng C 32: 303–309.

[mbt212340-bib-0003] Aulenta, F. , Rossetti, S. , Amalfitano, S. , Majone, M. , and Tandoi, V. (2013) Conductive magnetite nanoparticles accelerate the microbial reductive dechlorination of trichloroethene by promoting interspecies electron transfer processes. ChemSusChem 6: 433–436. 23401476 10.1002/cssc.201200748

[mbt212340-bib-0004] Beech, I.B. , and Sunner, J. (2004) Biocorrosion: towards understanding interactions between biofilms and metals. Curr Opin Biotechnol 15: 181–186. 15193324 10.1016/j.copbio.2004.05.001

[mbt212340-bib-0005] Beese‐Vasbender, P.F. , Grote, J.P. , Garrelfs, J. , Stratmann, M. , and Mayrhofer, K.J. (2015) Selective microbial electrosynthesis of methane by a pure culture of a marine lithoautotrophic archaeon. Bioelectrochemistry 102: 50–55. 25486337 10.1016/j.bioelechem.2014.11.004

[mbt212340-bib-0006] Bond, D.R. , and Lovley, D.R. (2003) Electricity production by *Geobacter sulfurreducens* attached to electrodes. Appl Environ Microbiol 69: 1548–1555. 12620842 10.1128/AEM.69.3.1548-1555.2003 PMC150094

[mbt212340-bib-0007] Brutinel, E.D. , and Gralnick, J.A. (2012) Shuttling happens: soluble flavin mediators of extracellular electron transfer in Shewanella. Appl Microbiol Biotechnol 93: 41–48. 22072194 10.1007/s00253-011-3653-0

[mbt212340-bib-0008] Castelle, C. , Guiral, M. , Malarte, G. , Ledgham, F. , Leroy, G. , Brugna, M. , and Giudici‐Orticoni, M.T. (2008) A new iron‐oxidizing/O_2_‐reducing supercomplex spanning both inner and outer membranes, isolated from the extreme acidophile *Acidithiobacillus ferrooxidans* . J Biol Chem 283: 25803–25811. 18632666 10.1074/jbc.M802496200 PMC3258861

[mbt212340-bib-0009] Daniels, L. , Belay, N. , Rajagopal, B.S. , and Weimer, P.J. (1987) Bacterial methanogenesis and growth from CO_2_ with elemental iron as the sole source of electrons. Science 237: 509–511. 17730323 10.1126/science.237.4814.509

[mbt212340-bib-0010] Deng, X. , Nakamura, R. , Hashimoto, K. , and Okamoto, A. (2015) Electron extraction from an extracellular electrode by *Desulfovibrio ferrophilus* strain IS5 without using hydrogen as an electron carrier. Electrochemistry 83: 529–531.

[mbt212340-bib-0011] Deutzmann, J.S. , Sahin, M. , and Spormann, A.M. (2015) Extracellular enzymes facilitate electron uptake in biocorrosion and bioelectrosynthesis. mBio 6: e00496–15. 25900658 10.1128/mBio.00496-15 PMC4453541

[mbt212340-bib-0012] Dickinson, W.H. , Caccavo, F. , Olesen, B. , and Lewandowski, Z. (1997) Ennoblement of stainless steel by the manganese‐depositing bacterium *Leptothrix discophora* . Appl Environ Microbiol 63: 2502–2506. 16535635 10.1128/aem.63.7.2502-2506.1997 PMC1389190

[mbt212340-bib-0013] Dinh, H.T. , Kuever, J. , Mussmann, M. , Hassel, A.W. , Stratmann, M. , and Widdel, F. (2004) Iron corrosion by novel anaerobic microorganisms. Nature 427: 829–832. 14985759 10.1038/nature02321

[mbt212340-bib-0014] Enning, D. , and Garrelfs, J. (2014) Corrosion of iron by sulfate‐reducing bacteria: new views of an old problem. Appl Environ Microbiol 80: 1226–1236. 24317078 10.1128/AEM.02848-13 PMC3911074

[mbt212340-bib-0015] Enning, D. , Venzlaff, H. , Garrelfs, J. , Dinh, H.T. , Meyer, V. , Mayrhofer, K. , *et al* (2012) Marine sulfate‐reducing bacteria cause serious corrosion of iron under electroconductive biogenic mineral crust. Environ Microbiol 14: 1772–1787. 22616633 10.1111/j.1462-2920.2012.02778.x PMC3429863

[mbt212340-bib-0016] Erable, B. , Féron, D. , and Bergel, A. (2012) Microbial catalysis of the oxygen reduction reaction for microbial fuel cells: a review. ChemSusChem 5: 975–987. 22615123 10.1002/cssc.201100836

[mbt212340-bib-0017] Gaines, R. (1910) Bacterial activity as a corrosive influence in the soil. J Ind Eng Chem 2: 128–130.

[mbt212340-bib-0018] Gieg, L.M. , Jack, T.R. , and Foght, J.M. (2011) Biological souring and mitigation in oil reservoirs. Appl Microbiol Biotechnol 92: 263–282. 21858492 10.1007/s00253-011-3542-6

[mbt212340-bib-0019] Gittel, A. , Seidel, M. , Kuever, J. , Galushko, A.S. , Cypionka, H. , and Könneke, M. (2010) *Desulfopila inferna* sp. nov., a sulfate‐reducing bacterium isolated from the subsurface of a tidal sand‐flat. Int J Syst Evol Microbiol 60: 1626–1630. 19717583 10.1099/ijs.0.015644-0

[mbt212340-bib-0020] Gorby, Y.A. , Yanina, S. , McLean, J.S. , Rosso, K.M. , Moyles, D. , Dohnalkova, A. , *et al* (2006) Electrically conductive bacterial nanowires produced by *Shewanella oneidensis* strain MR‐1 and other microorganisms. Proc Natl Acad Sci USA 103: 11358–11363. 16849424 10.1073/pnas.0604517103 PMC1544091

[mbt212340-bib-0021] Gralnick, J.A. , and Newman, D.K. (2007) Extracellular respiration. Mol Microbiol 65: 1–11. 17581115 10.1111/j.1365-2958.2007.05778.x PMC2804852

[mbt212340-bib-0022] Gregory, K.B. , Bond, D.R. , and Lovley, D.R. (2004) Graphite electrodes as electron donors for anaerobic respiration. Environ Microbiol 6: 596–604. 15142248 10.1111/j.1462-2920.2004.00593.x

[mbt212340-bib-0023] Hamilton, W.A. (2003) Microbially influenced corrosion as a model system for the study of metal microbe interactions: A unifying electron transfer hypothesis. Biofouling 19: 65–76. 14618690 10.1080/0892701021000041078

[mbt212340-bib-0024] Hardy, J.A. (1983) Utilisation of cathodic hydrogen by sulphate‐reducing bacteria. Br Corros J 18: 190–193.

[mbt212340-bib-0025] Hernandez, M.E. , and Newman, D.K. (2001) Extracellular electron transfer. Cell Mol Life Sci 58: 1562–1571. 11706984 10.1007/PL00000796 PMC11337281

[mbt212340-bib-0026] Hochella, M.F., Jr , Lower, S.K. , Maurice, P.A. , Penn, R.L. , Sahai, N. , Sparks, D.L. , and Twining, B.S. (2008) Nanominerals, mineral nanoparticles, and Earth systems. Science 319: 1631–1635. 18356515 10.1126/science.1141134

[mbt212340-bib-0027] Iino, T. , Ito, K. , Wakai, S. , Tsurumaru, H. , Ohkuma, M. , and Harayama, S. (2015) Iron corrosion induced by nonhydrogenotrophic nitrate‐reducing *Prolixibacter* sp. strain MIC1‐1. Appl Environ Microbiol 81: 1839–1846. 25548048 10.1128/AEM.03741-14 PMC4325155

[mbt212340-bib-0028] Jiang, J. , and Kappler, A. (2008) Kinetics of microbial and chemical reduction of humic substances: implications for electron shuttling. Environ Sci Technol 42: 3563–3569. 18546690 10.1021/es7023803

[mbt212340-bib-0029] Kato, S. (2015) Biotechnological aspects of microbial extracellular electron transfer. Microbes Environ 30: 133–139. 26004795 10.1264/jsme2.ME15028 PMC4462922

[mbt212340-bib-0030] Kato, S. , Kai, F. , Nakamura, R. , Watanabe, K. , and Hashimoto, K. (2010) Respiratory interactions of soil bacteria with (semi)conductive iron‐oxide minerals. Environ Microbiol 12: 3114–3123. 20561016 10.1111/j.1462-2920.2010.02284.x

[mbt212340-bib-0031] Kato, S. , Hashimoto, K. , and Watanabe, K. (2012a) Microbial interspecies electron transfer via electric currents through conductive minerals. Proc Natl Acad Sci USA 109: 10042–10046. 22665802 10.1073/pnas.1117592109 PMC3382511

[mbt212340-bib-0032] Kato, S. , Hashimoto, K. , and Watanabe, K. (2012b) Methanogenesis facilitated by electric syntrophy via (semi)conductive iron‐oxide minerals. Environ Microbiol 14: 1646–1654. 22004041 10.1111/j.1462-2920.2011.02611.x

[mbt212340-bib-0033] Kato, S. , Hashimoto, K. , and Watanabe, K. (2013) Iron‐oxide minerals affect extracellular electron‐transfer paths of *Geobacter* spp. Microbes Environ 28: 141–148. 23363619 10.1264/jsme2.ME12161 PMC4070692

[mbt212340-bib-0034] Kato, S. , Yumoto, I. , and Kamagata, Y. (2015) Isolation of acetogenic bacteria that induce biocorrosion by utilizing metallic iron as the sole electron donor. Appl Environ Microbiol 81: 67–73. 25304512 10.1128/AEM.02767-14 PMC4272740

[mbt212340-bib-0035] Kim, B.H. , Lim, S.S. , Daud, W.R. , Gadd, G.M. , and Chang, I.S. (2015) The biocathode of microbial electrochemical systems and microbially‐influenced corrosion. Bioresour Technol 190: 390–401. 10.1016/j.biortech.2015.04.084 25976915

[mbt212340-bib-0036] King, R.A. , and Miller, J.D.A. (1971) Corrosion by the sulphate‐reducing bacteria. Nature 233: 491–492. 16063454 10.1038/233491a0

[mbt212340-bib-0037] Lee, W. , Lewandowski, Z. , Nielsen, P.H. , and Hamilton, W.A. (1995) Role of sulfate‐reducing bacteria in corrosion of mild steel: A review. Biofouling 8: 165–194.

[mbt212340-bib-0038] Lin, K.S. , Chang, N.B. , and Chuang, T.D. (2008) Fine structure characterization of zero‐valent iron nanoparticles for decontamination of nitrites and nitrates in wastewater and groundwater. Sci Technol Adv Mater 9: 025015. 10.1088/1468-6996/9/2/025015 PMC5099747 27877990

[mbt212340-bib-0039] Little, B. , Staehle, R. , and Davis, R. (2001) Fungal influenced corrosion of post‐tensioned cables. Int Biodeterior Biodegrad 47: 71–77.

[mbt212340-bib-0040] Logan, B.E. , and Rabaey, K. (2012) Conversion of wastes into bioelectricity and chemicals by using microbial electrochemical technologies. Science 337: 686–690. 22879507 10.1126/science.1217412

[mbt212340-bib-0041] Lovley, D.R. (2008) The microbe electric: conversion of organic matter to electricity. Curr Opin Biotechnol 19: 564–571. 19000760 10.1016/j.copbio.2008.10.005

[mbt212340-bib-0042] Mand, J. , Park, H.S. , Jack, T.R. , and Voordouw, G. (2014) The role of acetogens in microbially influenced corrosion of steel. Front Microbiol 5: 268. 24917861 10.3389/fmicb.2014.00268 PMC4043135

[mbt212340-bib-0043] Marsili, E. , Baron, D.B. , Shikhare, I.D. , Coursolle, D. , Gralnick, J.A. , and Bond, D.R. (2008) *Shewanella* secretes flavins that mediate extracellular electron transfer. Proc Natl Acad Sci USA 105: 3968–3973. 18316736 10.1073/pnas.0710525105 PMC2268775

[mbt212340-bib-0044] Mori, K. , Tsurumaru, H. , and Harayama, S. (2010) Iron corrosion activity of anaerobic hydrogen‐consuming microorganisms isolated from oil facilities. J Biosci Bioeng 110: 426–430. 20547365 10.1016/j.jbiosc.2010.04.012

[mbt212340-bib-0045] Nakamura, R. , Kai, F. , Okamoto, A. , Newton, G.J. , and Hashimoto, K. (2009) Self‐constructed electrically conductive bacterial networks. Angew Chem Int Ed 48: 508–511. 10.1002/anie.200804750 19072958

[mbt212340-bib-0046] Nakamura, R. , Takashima, T. , Kato, S. , Takai, K. , Yamamoto, M. , and Hashimoto, K. (2010a) Electrical current generation across a black smoker chimney. Angew Chem Int Ed 49: 7692–7694. 10.1002/anie.201003311 20839203

[mbt212340-bib-0047] Nakamura, R. , Okamoto, A. , Tajima, N. , Newton, G.J. , Kai, F. , Takashima, T. , and Hashimoto, K. (2010b) Biological iron‐monosulfide production for efficient electricity harvesting from a deep‐sea metal‐reducing bacterium. Chembiochem 11: 643–645. 20146276 10.1002/cbic.200900775

[mbt212340-bib-0048] Nevin, K.P. , Hensley, S.A. , Franks, A.E. , Summers, Z.M. , Ou, J. , Woodard, T.L. , *et al* (2011) Electrosynthesis of organic compounds from carbon dioxide is catalyzed by a diversity of acetogenic microorganisms. Appl Environ Microbiol 77: 2882–2886. 21378039 10.1128/AEM.02642-10 PMC3126412

[mbt212340-bib-0049] Nishio, K. , Nakamura, R. , Lin, X. , Konno, T. , Ishihara, K. , Nakanishi, S. , and Hashimoto, K. (2013) Extracellular electron transfer across bacterial cell membranes via a cytocompatible redox‐active polymer. Chemphyschem 14: 2159–2163. 23630181 10.1002/cphc.201300117

[mbt212340-bib-0050] Pirbadian, S. , Barchinger, S.E. , Leung, K.M. , Byun, H.S. , Jangir, Y. , Bouhenni, R.A. , *et al* (2014) *Shewanella oneidensis* MR‐1 nanowires are outer membrane and periplasmic extensions of the extracellular electron transport components. Proc Natl Acad Sci USA 111: 12883–12888. 25143589 10.1073/pnas.1410551111 PMC4156777

[mbt212340-bib-0051] Rabaey, K. , and Rozendal, R.A. (2010) Microbial electrosynthesis – revisiting the electrical route for microbial production. Nat Rev Microbiol 8: 706–716. 20844557 10.1038/nrmicro2422

[mbt212340-bib-0052] Rabaey, K. , Boon, N. , Siciliano, S.D. , Verhaege, M. , and Verstraete, W. (2004) Biofuel cells select for microbial consortia that self‐mediate electron transfer. Appl Environ Microbiol 70: 5373–5382. 15345423 10.1128/AEM.70.9.5373-5382.2004 PMC520914

[mbt212340-bib-0053] Reguera, G. , McCarthy, K.D. , Mehta, T. , Nicoll, J.S. , Tuominen, M.T. , and Lovley, D.R. (2005) Extracellular electron transfer via microbial nanowires. Nature 435: 1098–1101. 15973408 10.1038/nature03661

[mbt212340-bib-0054] Richter, K. , Schicklberger, M. , and Gescher, J. (2012) Dissimilatory reduction of extracellular electron acceptors in anaerobic respiration. Appl Environ Microbiol 78: 913–921. 22179232 10.1128/AEM.06803-11 PMC3273014

[mbt212340-bib-0055] Rosenbaum, M. , Aulenta, F. , Villano, M. , and Angenent, L.T. (2011) Cathodes as electron donors for microbial metabolism: which extracellular electron transfer mechanisms are involved? Bioresour Technol 102: 324–333. 20688515 10.1016/j.biortech.2010.07.008

[mbt212340-bib-0056] Shi, L. , Squier, T.C. , Zachara, J.M. , and Fredrickson, J.K. (2007) Respiration of metal (hydr)oxides by *Shewanella* and *Geobacter*: A key role for multihaem *c*‐type cytochromes. Mol Microbiol 65: 12–20. 17581116 10.1111/j.1365-2958.2007.05783.x PMC1974784

[mbt212340-bib-0057] Stams, A.J. , de Bok, F.A. , Plugge, C.M. , van Eekert, M.H. , Dolfing, J. , and Schraa, G. (2006) Exocellular electron transfer in anaerobic microbial communities. Environ Microbiol 8: 371–382. 16478444 10.1111/j.1462-2920.2006.00989.x

[mbt212340-bib-0058] Suflita, J.M. , Phelps, T.J. , and Little, B. (2008) Carbon dioxide corrosion and acetate: a hypothesis on the influence of microorganisms. Corros Sci 64: 854–859.

[mbt212340-bib-0059] Summers, Z.M. , Fogarty, H.E. , Leang, C. , Franks, A.E. , Malvankar, N.S. , and Lovley, D.R. (2010) Direct exchange of electrons within aggregates of an evolved syntrophic coculture of anaerobic bacteria. Science 330: 1413–1415. 21127257 10.1126/science.1196526

[mbt212340-bib-0060] Thrash, J.C. , and Coates, J.D. (2008) Review: direct and indirect electrical stimulation of microbial metabolism. Environ Sci Technol 42: 3921–3931. 18589946 10.1021/es702668w

[mbt212340-bib-0061] Uchiyama, T. , Ito, K. , Mori, K. , Tsurumaru, H. , and Harayama, S. (2010) Iron‐corroding methanogen isolated from a crude‐oil storage tank. Appl Environ Microbiol 76: 1783–1788. 20118376 10.1128/AEM.00668-09 PMC2838011

[mbt212340-bib-0062] Usher, K.M. , Kaksonen, A.H. , and MacLeod, I.D. (2014) Marine rust tubercles harbour iron corroding archaea and sulphate reducing bacteria. Corros Sci 83: 189–197.

[mbt212340-bib-0063] Venzlaff, H. , Enning, D. , Srinivasan, J. , Mayrhofer, K. , Hassel, A.W. , Widdel, F. , and Stratmann, M. (2013) Accelerated cathodic reaction in microbial corrosion of iron due to direct electron uptake by sulfate‐reducing bacteria. Corros Sci 66: 88–96.

[mbt212340-bib-0064] Videla, H.A. , and Herrera, L.K. (2005) Microbiologically influenced corrosion: looking to the future. Int Microbiol 8: 169–180. 16200495

[mbt212340-bib-0065] Wakai, S. , Ito, K. , Iino, T. , Tomoe, Y. , Mori, K. , and Harayama, S. (2014) Corrosion of iron by iodide‐oxidizing bacteria isolated from brine in an iodine production facility. Microb Ecol 68: 519–527. 24863130 10.1007/s00248-014-0438-x

[mbt212340-bib-0066] Watanabe, K. , Manefield, M. , Lee, M. , and Kouzuma, A. (2009) Electron shuttles in biotechnology. Curr Opin Biotechnol 20: 633–641. 19833503 10.1016/j.copbio.2009.09.006

[mbt212340-bib-0067] Weber, K.A. , Achenbach, L.A. , and Coates, J.D. (2006) Microorganisms pumping iron: anaerobic microbial iron oxidation and reduction. Nat Rev Microbiol 4: 752–764. 16980937 10.1038/nrmicro1490

[mbt212340-bib-0068] Wolzogen Kühr, C.A.H. (1961) Unity of anaerobic and aerobic iron corrosion process in the soil. Corrosion 17: 293–299.

[mbt212340-bib-0069] Xu, D. , Li, Y. , Song, F. , and Gu, T. (2013) Laboratory investigation of microbiologically influenced corrosion of C1018 carbon steel by nitrate reducing bacterium *Bacillus licheniformis* . Corros Sci 77: 385–390.

[mbt212340-bib-0070] Zhang, P. , Xu, D. , Li, Y. , Yang, K. , and Gu, T. (2015) Electron mediators accelerate the microbiologically influenced corrosion of 304 stainless steel by the *Desulfovibrio vulgaris* biofilm. Bioelectrochemistry 101: 14–21. 25023048 10.1016/j.bioelechem.2014.06.010

